# Utilizing Spatial Demographic and Life History Variation to Optimize Sustainable Yield of a Temperate Sex-Changing Fish

**DOI:** 10.1371/journal.pone.0024580

**Published:** 2011-09-06

**Authors:** Scott L. Hamilton, Jono R. Wilson, Tal Ben-Horin, Jennifer E. Caselle

**Affiliations:** 1 Marine Science Institute, University of California Santa Barbara, Santa Barbara, California, United States of America; 2 Moss Landing Marine Laboratories, Moss Landing, California, United States of America; 3 Bren School of Environmental Science and Management, University of California Santa Barbara, Santa Barbara, California, United States of America; University of California San Diego, United States of America

## Abstract

Fish populations vary geographically in demography and life history due to environmental and ecological processes and in response to exploitation. However, population dynamic models and stock assessments, used to manage fisheries, rarely explicitly incorporate spatial variation to inform management decisions. Here, we describe extensive geographic variation in several demographic and life history characteristics (e.g., size structure, growth, survivorship, maturation, and sex change) of California sheephead (*Semicossyphus pulcher*), a temperate rocky reef fish targeted by recreational and commercial fisheries. Fish were sampled from nine locations throughout southern California in 2007–2008. We developed a dynamic size and age-structured model, parameterized separately for each location, to assess the potential cost or benefit in terms of fisheries yield and conservation objectives of changing minimum size limits and/or fishing mortality rates (compared to the status quo). Results indicate that managing populations individually, with location-specific regulations, could increase yield by over 26% while maintaining conservative levels of spawning biomass. While this local management approach would be challenging to implement in practice, we found statistically similar increases in yield could be achieved by dividing southern California into two separate management regions, reflecting geographic similarities in demography. To maximize yield, size limits should be increased by 90 mm in the northern region and held at current levels in the south. We also found that managing the fishery as one single stock (the status quo), but with a size limit 50 mm greater than the current regulations, could increase overall fishery yield by 15%. Increases in size limits are predicted to enhance fishery yield and may also have important ecological consequences for the predatory role of sheephead in kelp forests. This framework for incorporating demographic variation into fisheries models can be exported generally to other species and may aid in identifying the appropriate spatial scales for fisheries management.

## Introduction

For harvested fish species, stock assessment models are commonly used to estimate current and virgin biomass and fishing mortality rates that will achieve a predetermined objective [1]–[3]. Traditional models have explored the impacts of growth, recruitment, and mortality on stock dynamics, but have rarely explicitly assessed stock status as a function of demographic and life history variation among populations or sub-populations. Often, key model parameters, such as growth rates and the timing of maturation, are drawn from a single location or averaged over multiple locations [4], [5]. Demographic variation, if included, is commonly relegated simply to error that propagates through the model [4], [6]–[7]. Therefore, most fish stocks are managed with a single minimum size limit and a universal fishing mortality rate regardless of the geographic distribution of the resource. For highly mobile species, this traditional approach is often valid. However, temperate rocky reef fish pose problems for traditional fisheries management because emerging evidence suggests that adults have relatively small home ranges [8], [9] and more limited larval exchange than previously thought [10], [11]. Temperate reef fishes may commonly exhibit plasticity in their demographic and life history traits, requiring a more localized approach to management [12]. By explicitly incorporating these sources of variation in fisheries models, and assigning distinct size or catch limits to different management regions, it may be possible to optimize yield while achieving sustainability-oriented objectives for the entire fishery.

It is well known that many species vary geographically in their ecology over a range of spatial scales [13]. Biogeographic variation in demography and life histories can occur naturally in response to changes in environmental conditions, such as temperature, habitat, prey availability, diet composition, and predation pressure [14]–[22]. Fish are often larger and grow faster in cooler waters, locations with high productivity or preferred prey, and fewer predators. Size-selective fishing pressure, by targeting larger and faster-growing individuals, has also been shown to alter demographic and life history traits, such as growth rates, reproductive output, longevity, and the timing of maturation and sex change [23]–[30]. Current patterns of spatial demographic and life history variation are likely influenced by environmental gradients, evolutionary history, and fishing pressure, making it a challenge to ascribe specific mechanisms to explain the patterns among locations. Despite challenges inherent in identifying the mechanisms, the impact of this variation can have severe consequences on local populations for which stock-wide regulations are not appropriately matched to the biological reality of the resource.

Spatial scales of intraspecific demographic variation have seldom been assessed for temperate reef fishes [but see 23,30–32], despite the economic importance of many of these species and the potential for significant geographic variation among populations. In contrast, a wealth of research has revealed extensive geographic variation in demographic and life history traits of coral reef fishes, due to temperature, habitat, and predation pressure [16], [17], [20], [21]. However, previous research on populations of California sheephead (*Semicossyphus pulcher*) along the Pacific coast of North America has found significant geographic structuring of life history traits caused by natural mortality rates, temperature, population density and sex ratios, prey availability and diet composition, and the history of exploitation [22], [29], [33]–[35].

California sheephead are large temperate wrasses that are common in kelp beds and rocky reefs from southern California through Baja California, Mexico. They are predators on sea urchins and other benthic invertebrates and play a critical role in regulating prey populations in kelp forests [34], [36], [37]. Home ranges are relatively small and individuals appear to show site fidelity over the course of a year [8], [38]. California sheephead are protogynous hermaphrodites and are capable of changing sex from female to male [33]. Important commercial and recreational fisheries exist for this species throughout its range [5], [39]. Commercial landings increased dramatically in southern California throughout the 1990s with the advent of a trap fishery for live caught fish [5]. Size and catch limits for commercial and recreational sectors were first implemented in 1999, prompted by high fishing mortality rates in a previously unregulated fishery. A stock assessment, based largely on data from the 1970s–1980s from relatively unfished locations, stressed the need for more current information on spatial variation in the status of different populations [5]. This information holds great importance to fisheries managers because size-selective harvesting has been shown to significantly alter life histories of the specific populations used in the stock assessment [29]. While the stock assessment acknowledged spatial variation in population parameters from past studies over large biogeographic scales [33], [35], ultimately the fishery model for managing the California fishery was parameterized with demographic and life history data from only one population in southern California; Santa Catalina Island, because limited information existed for other areas.

Recent fisheries models have highlighted the potential vulnerability of protogynous species to overexploitation, because size-selective harvest is commonly biased towards males and fishing can therefore drastically reduce reproductive rates and fertilization success, compared to dioecious (separate sex) species [40]–[42]. For California sheephead, further applications of the model used in the stock assessment emphasized how size-selective harvest, life history strategies, and sex change rules can affect stock dynamics and spawning-per-recruit measures [42], [43]. The results of these studies suggest that for protogynous species in particular, the risk of population crashes can be assessed by explicitly incorporating spatial demographic and life history variation into fisheries models. Ultimately, more localized fisheries management, with size limits tuned to the biological characteristics (i.e., growth rates, timing of maturation and sex change) of different populations or regions may help to maintain spawning biomass and improve yield for the fishery as a whole.

In this study, we describe geographic variation in size structure, demography, and life histories of California sheephead from nine locations (from samples collected in 2007–2008) throughout southern California, where the fishery is currently managed. While previous studies have documented geographic variation among California sheephead populations [22], [33], [35], those studies have focused on comparisons over large geographic scales, between a few southern California, U.S.A. and Baja California, Mexico populations. In addition, within southern California those studies examined either relatively unfished populations in the 1970s–1980s [33], [35] or populations during the year of peak fishery landings in 1998 [22], [29], prior to the implementation of size and catch limits. Here, we update the population status of California sheephead across southern California and describe a general approach for incorporating demographic and life history variation into area-based management strategies. We used population-based simulations, with parameters drawn from each of nine locations, to estimate relative spawning stock biomass (calculated as total no. eggs) and yield as a function of fishing pressure. We predict the optimal size limit and fishing mortality rate for each population that will allow for long-term population persistence while maximizing yield. Then, to gauge the potential biological importance of demographic and life history variation for fisheries management, we compare differences in yield between simulations where regulations are set locally (i.e., population-specific), regionally (i.e., populations grouped into a subset of regions), globally (i.e., assumption of one stock), or fixed at their current level. Ultimately, we find that dividing southern California into two separate management regions may benefit the fishery and aid the long-term sustainability of this species.

## Materials and Methods

### Study locations, collections, and measurements

**Figure 1:**
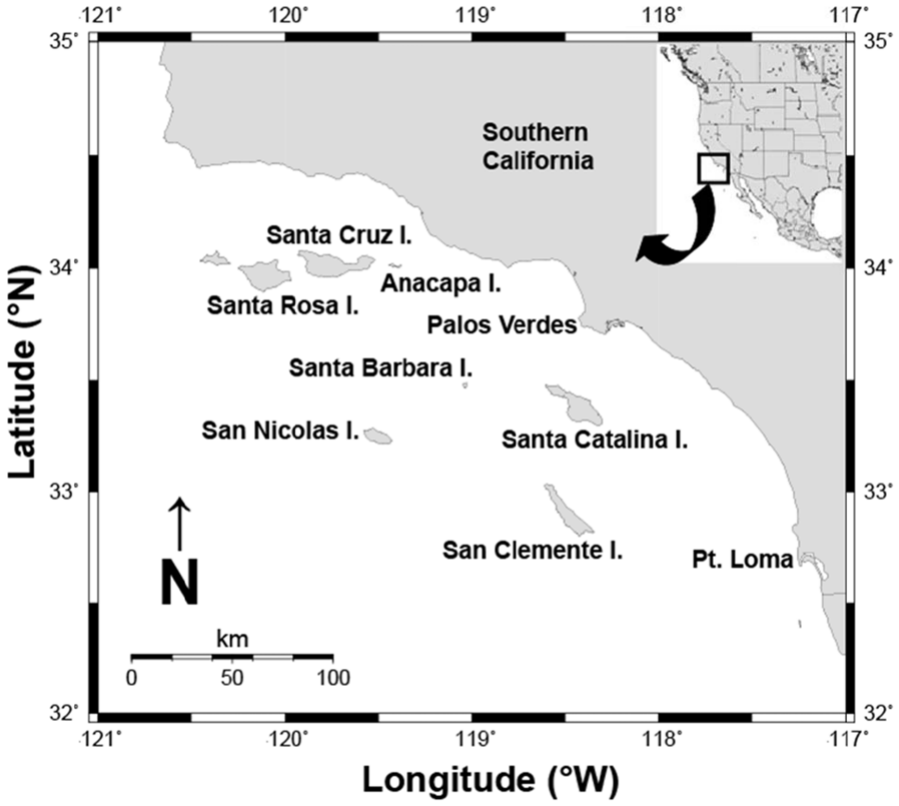
Map of southern California, showing the nine island and mainland populations of California sheephead sampled for the current study.

This study was approved by the Institutional Animal Care and Use Committee at the University of California Santa Barbara (Permit Number: 729) and care was taken to minimize the suffering of animals. We collected samples of California sheephead from nine discrete locations in southern California, during June-September of 2007–2008 (Fig. 1). Individual California sheephead were collected by spear similar to methods reported in ref. [33], [35]. To ensure an unbiased collection of particular size classes we pursued and speared each fish encountered, regardless of size or sex, before proceeding to another individual (*n* = 44–76 fish per site; Table 1). On occasion we also collected fish with hook and line gear or baited fish traps. We recorded the standard length (SL, mm), total length (TL, mm), wet weight (g), and coloration (male or female color phases) of each individual. Sex was determined macroscopically by observing the color, texture, and appearance of the gonads or by examining unripe ovaries for the presence of maturing eggs [as in ref. 35]. Further confirmation of sex occurred through histological preparations of gonad samples [44]. Because reproductive activity begins in May [33], [35] and our sampling occurred during or after this month, we encountered little difficulty in separating fish into immature, female, and male sexual classes. All transitional individuals (i.e., in the process of sex change and identified by intermediate morphological coloration and gonad histology) were categorized as male for presentation and analysis.

**Table 1:** Sample sizes, demographic, and life history information for California sheephead from nine populations studied in southern California as well as those calculated for all populations in each region and for all populations in the study.

Population	*N*	Mean size (SL, ±SE)	*L_inf_*	*K*	Maturation (years; SL)	Sex change (years; SL)	Survivorship (±SE)	Max age (years)
Santa Rosa I.	44	354±9.3	543.7	0.171	A_50♀_ = 3.9 L_50♀_ = 270	A_50♂_ = 8.9 L_50♂_ = 413	0.845±0.037	20
Santa Cruz I.	76	311±10.7	548.5	0.154	A_50♀_ = 4.1 L_50♀_ = 268	A_50♂_ = 11.3 L_50♂_ = 419	0.834±0.036	29
Anacapa I.	59	326±10.7	540.0	0.150	A_50♀_ = 4.9 L_50♀_ = 290	A_50♂_ = 11.1 L_50♂_ = 433	0.810±0.017	17
San Nicolas I.	71	392±9.5	639.1	0.124	A_50♀_ = 4.8 L_50♀_ = 283	A_50♂_ = 8.4 L_50♂_ = 408	0.812±0.026	20
Santa Barbara I.	56	352±9.9	571.6	0.134	A_50♀_ = 4.4 L_50♀_ = 261	A_50♂_ = 10.0 L_50♂_ = 413	0.816±0.016	17
Palos Verdes	46	243±7.2	403.8	0.175	A_50♀_ = 4.9 L_50♀_ = 236	A_50♂_ = 7.7 L_50♂_ = 299	0.736±0.034	10
Santa Catalina I.	44	234±4.7	305.3	0.245	A_50♀_ = 4.0 L_50♀_ = 209	A_50♂_ = 6.2 L_50♂_ = 230	0.699±0.032	11
San Clemente I.	50	244±7.1	396.9	0.128	A_50♀_ = 4.9 L_50♀_ = 175	A_50♂_ = 7.5 sL_50♂_ = 241	0.694±0.019	14
Point Loma	53	273±9.5	451.3	0.169	A_50♀_ = 4.7 L_50♀_ = 253	A_50♂_ = 7.8 L_50♂_ = 320	0.786±0.031	15
Regional and Global population parameters								
Zone 1 (north)	282	346±5.1	361.5	0.196	A_50♀_ = 4.4 L_50♀_ = 273	A_50♀_ = 9.6 L_50♀_ = 414	0.820±0.020	29
Zone 2 (south)	188	251±3.9	569.6	0.146	A_50♀_ = 4.8 L_50♀_ = 217	A_50♀_ = 7.3 L_50♀_ = 269	0.696±0.023	15
Global (all pop.)	470	309±4.1	557.7	0.126	A_50♀_ = 4.6 L_50♀_ = 242	A_50♀_ = 8.7 L_50♀_ = 403	0.803±0.020	29

Sizes are standard length (SL) in mm. All other variables are defined in the methods.

The first two dorsal spines were removed, cleaned, and frozen for age determination using methods reported in ref. [29] and modified from ref. [33]. We prepared cross sections of the 1^st^ dorsal spine for aging (occasionally the 2^nd^ spine was used if the 1^st^ spine yielded poor resolution of annual bands). We used a Dremel® tool to cut thin sections by removing the base and top of each spine. Sections of spine were embedded vertically in Crystalbond® (Electron Microscopy Sciences) and polished using a lapping wheel (South Bay Technologies) with 15, 9, and 3 µm polishing films to improve ring clarity. Two observers counted annual rings using an image analysis system (Image Pro 6.3) connected to a compound microscope at 40× power.

### Demographic and life history analysis

We assessed spatial differences in the sizes of each sex using ANOVA. To examine spatial differences in lifetime growth trajectories, we fit von Bertalanffy growth functions (VBGF) to the size (SL) at age data from each focal population using least squares techniques and the following equation,
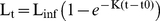
(1)


where L_t_ equals the predicted length at age, L_inf_ is the predicted maximum asymptotic length, *K* is the coefficient of growth (or how quickly individuals approach the asymptotic length), t equals age, and t_0_ is the theoretical length at which the fish is age zero. We fixed t_0_ at zero for estimating L_inf_ and *K*, following ref. [20]. We then extracted the VBGF parameters from the best-fit model to generate growth curves for each location. We used maximum likelihood techniques to estimate the 95% confidence bounds around the best-fit VBGF parameter values following ref. [45] in R [46].

We used data on size, age, and sex to estimate spatial differences in the timing of maturation and sex change among the nine locations. The size or age at maturity was defined as the size or age at which females began to predominate over immatures in the population (i.e., L_50♀_ or A_50♀_; length or age at 50% mature female). Comparably, the size or age at sex change was defined as the size or age at which males began to predominate over mature females in the population (i.e., L_50♂_ or A_50♂_; length or age at 50% male). We used logistic regression to determine the predicted timing of maturation and sexual transformation of each population. Statistical analyses were performed in JMP 8.0.

We used age-based catch curves to estimate mortality rates (*Z*) following standard fisheries methods [e.g., 20, 47]. Total instantaneous mortality rates (*Z*) were calculated using log-linear regressions of the age-frequency data (*Z* = regression slope), excluding fish younger than the modal age (age frequencies peaked between 4–6 years). Estimates of annual survivorship (*S*) were then calculated according to the equation,
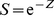
(2)


following ref. [48].

### Fishery Population Model

We built separate size- and age-structured population dynamics models for each of the nine locations for which data were collected during the course of this study. The underlying model is similar in format to those described in refs. [49], [50]. Each population is considered closed with respect to recruitment and migration. We use 20 age classes and a plus group. We assume no sperm limitation due to the prevalence of sneaker males and the potentially high reproductive capabilities of individual adult males [5], [50]. The proportion of mature females at a given size was calculated as the product of the proportions predicted by a logistic function for size at maturity and another for size at sex change [50]. We defined fecundity as total annual egg production estimated as a function of total body weight and based on published studies [33], [50], [51]. We assume the size-fecundity relationship to be constant across sites [33].

Growth rates among populations were described by the von Bertalanffy equation at each location, as discussed previously. Fishery selectivity is determined by a minimum size limit and is modeled as the probability that an individual of a given age is greater than the minimum size limit as described by a logistic equation. The commercial minimum size limit of 30 cm TL (equivalent to 273 mm SL) was used for all analyses. Using mortality (*Z*) as described above, we found that particular *Z* values were below the natural mortality (*M*) rate of 0.2 assumed (with high uncertainty) in the 2004 stock assessment [5]. Although it can be assumed that natural mortality is variable through space, we are currently unable to separate natural from fishing mortality, and thus we assume that natural mortality is 0.1 at all sites and is independent of age and time. Therefore, we calculate current fishing mortality to be the difference between *Z* and *M* at each site (*F* = *Z* − *M*). Although this is a simplification, our results are meant to be qualitatively informative and are relative to the base case scenario in which we also use a natural mortality rate of 0.1.

Recruitment was modeled with a Beverton-Holt stock recruitment function with log-normal random deviations of 0.6 [5]. At equilibrium this is defined by:
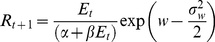
(3)


where α and β are parameters of the Beverton-Holt spawner-recruit curve and *w* is a log-normally distributed random variable with mean zero and standard deviation *σ_w_*.
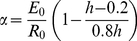
(4)

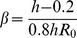
(5)


where *E_0_* is the egg production in the absence of fishing mortality, *R_0_* is the recruitment in the absence of fishing mortality, and *h* is the steepness which describes the sensitivity of recruitment to spawning stock biomass (SSB). We set steepness equal to 0.7, to coincide with the value estimated in the 2004 stock assessment [5]. The steepness parameter is defined as:
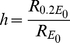
(6)


Following ref. [52].

The starting conditions for the age groups are:
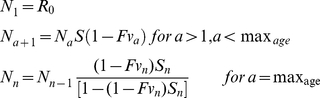
(7)


where *S* is survival from natural mortality, *F* is the instantaneous fishing mortality rate, *v_a_* is the vulnerability to fishing of fish aged *a*, and *R_0_* is the recruitment in year 1. Egg production and recruitment in the absence of fishing mortality are related as follows:
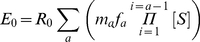
(8)


where *m_a_* is the fraction of the population of age *a* which are mature females and *f_a_* is the number of eggs per mature female of age *a*. The number of individuals of each age thereafter is defined as:
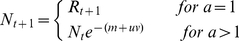
(9)


where *R_t_* is the recruitment in year t. The catch (expressed in biomass) is defined as:
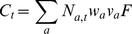
(10)


while spawning stock biomass (SSB) is defined as:

B_v_ = 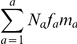


Our objective was to explore tradeoffs in potential yield and sustainability by adjusting location-specific minimum size limits and fishing mortality rates. The model is intended to demonstrate the relative effects of demographic variation on management objectives in the fishery. We do not fit the model to historical catch and effort data, nor do we make an attempt to estimate current or virgin biomass levels. Rather, each of the nine locations begins at equilibrium levels of abundance, and interactions between fishing mortality rates and location-specific demographics influence the dynamics and the outcomes of our model. All populations were initialized with a stable age distribution in the absence of fishing mortality starting with 1000 age zero individuals in year zero. Virgin SSB estimates were calculated as the product of the year zero population age structure, fecundity and maturity ogives. We simulated population dynamics at each site for 100 years under variable minimum size limits and fishing mortality rates. Yield and SSB estimates at year 100 were used as a proxy for equilibrium conditions for each scenario. We defined equilibrium SSB levels as the proportion of spawning stock biomass between the median equilibrium value and the unfished level. We introduced stochasticity through log-normal standard deviates of recruitment which were consistent across locations (eq. 1). No attempt is made to adjust sex-changing functions through time, in relation to altered sex ratios or through size- and sex-selective fishing mortality, although this could be done in the future.

We ran 1000 Monte Carlo simulations for every plausible combination of minimum size limit (200 – 400 mm) and fishing mortality rate (*F*; 0 – 1.5) at each location. In order to maintain sustainability objectives we set a minimum stock size threshold (MSST) equal to 10% of unfished spawning stock biomass [53], [54]. If any combination of minimum size limit and fishing mortality rate in any single year of a given simulation caused the population to drop below this threshold in more than 5% of the simulations, that combination of minimum size limit and fishing mortality rate was considered inappropriate for use in the sustainable management of the fishery.

We then calculated the sustainability-oriented maximum potential yield as the median catch biomass at year 100 over the 1000 simulations for the optimal combination of minimum size limit and fishing mortality rate, eliminating those combinations where threshold levels of biomass violated our sustainability objective. We calculated this sustainability-oriented yield for each of the nine locations as well as the cumulative fishery yield for all locations combined. We used these values to answer the following questions:

1. What is the location-specific yield benefit to:
  - optimizing minimum size limits, while keeping the current fishing mortality rates constant?
  - optimizing fishing mortality rates, while keeping the current minimum size limit constant?
  - optimizing both minimum size limits and fishing mortality rates simultaneously?
2. What is the overall fishery yield benefit:
  - under local management (i.e., setting different optimal minimum size limits and fishing mortality rates for each population) relative to the current management regulations?
  - after optimizing size limits and fishing mortality rates under the assumption of one (i.e., global management) or two (i.e. regional management) separate stocks in southern California, relative to the current management regulations?

## Results

### Geographic variation in demography and life histories

**Figure 2:**
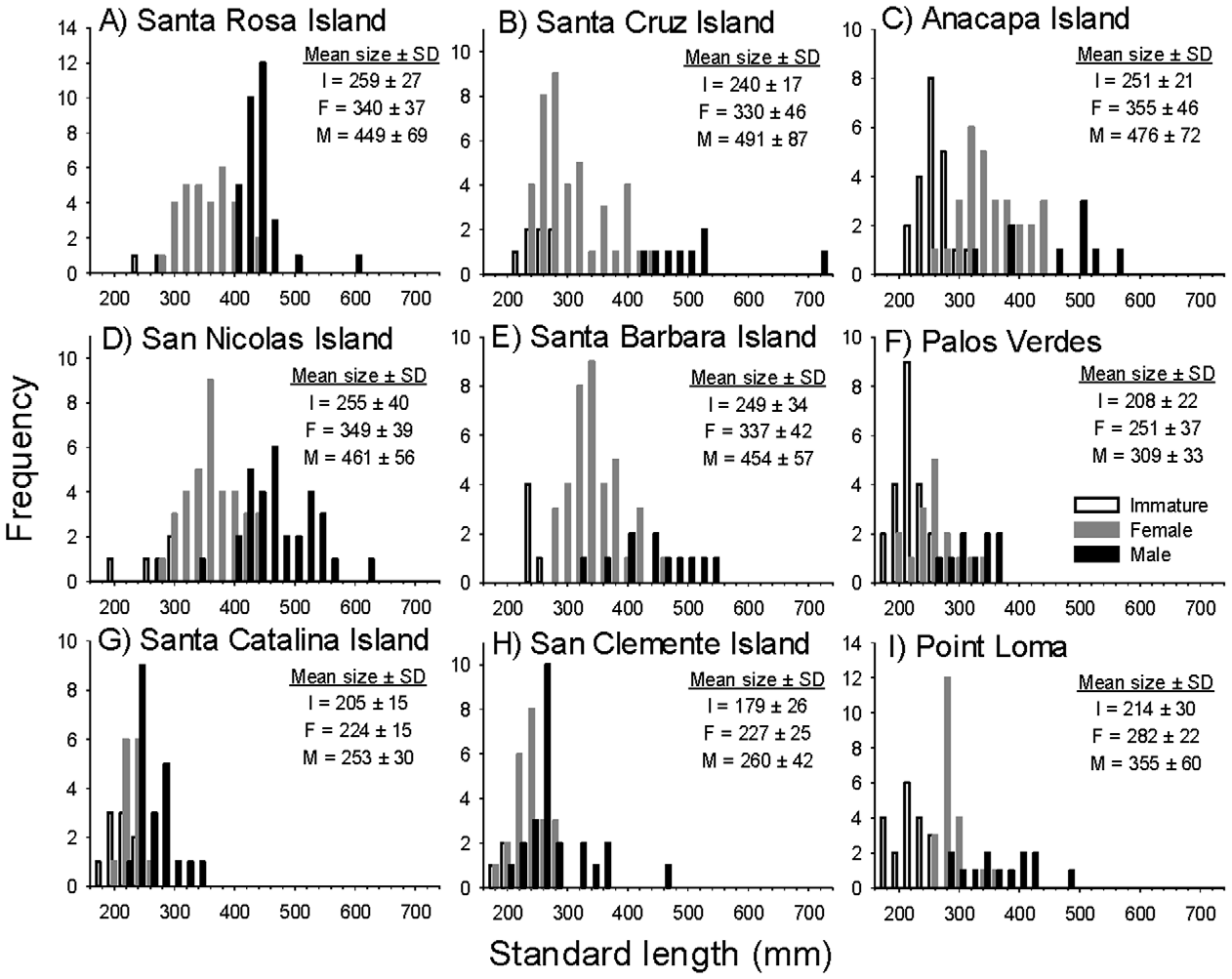
Size frequency distributions of nine populations of California sheephead sampled throughout southern California. Inset legends show mean size (SL, mm) ±1 SD for immature (white bars, I), female (gray bars, F) and male (black bars, M) sexual classes.

Size frequency distributions of the nine California sheephead populations differed significantly (ANOVA, *F_8, 489_* = 35.0, *P*<0.0001) and generally followed a latitudinal pattern, with larger fish present in cooler waters of the northern Channel Islands (Santa Rosa, Santa Cruz, Anacapa, San Nicolas and Santa Barbara islands), than the mainland (Palos Verdes and Point Loma), and the southern Channel Islands (Santa Catalina and San Clemente) (Table 1; Fig. 2). Size frequencies of the different sexual classes also differed significantly among the sampled locations (ANOVA, Immatures: *F_8, 101_* = 10.7, *P*<0.0001; Females: *F_8, 221_* = 42.6, *P*<0.0001; Males: *F_8, 119_* = 42.9, *P*<0.0001) and followed similar spatial patterns, with sizes of each sexual class being largest at the northern Channel Islands, intermediate in size at the two mainland locations, and smallest at the southern Channel Islands.

**Figure 3:**
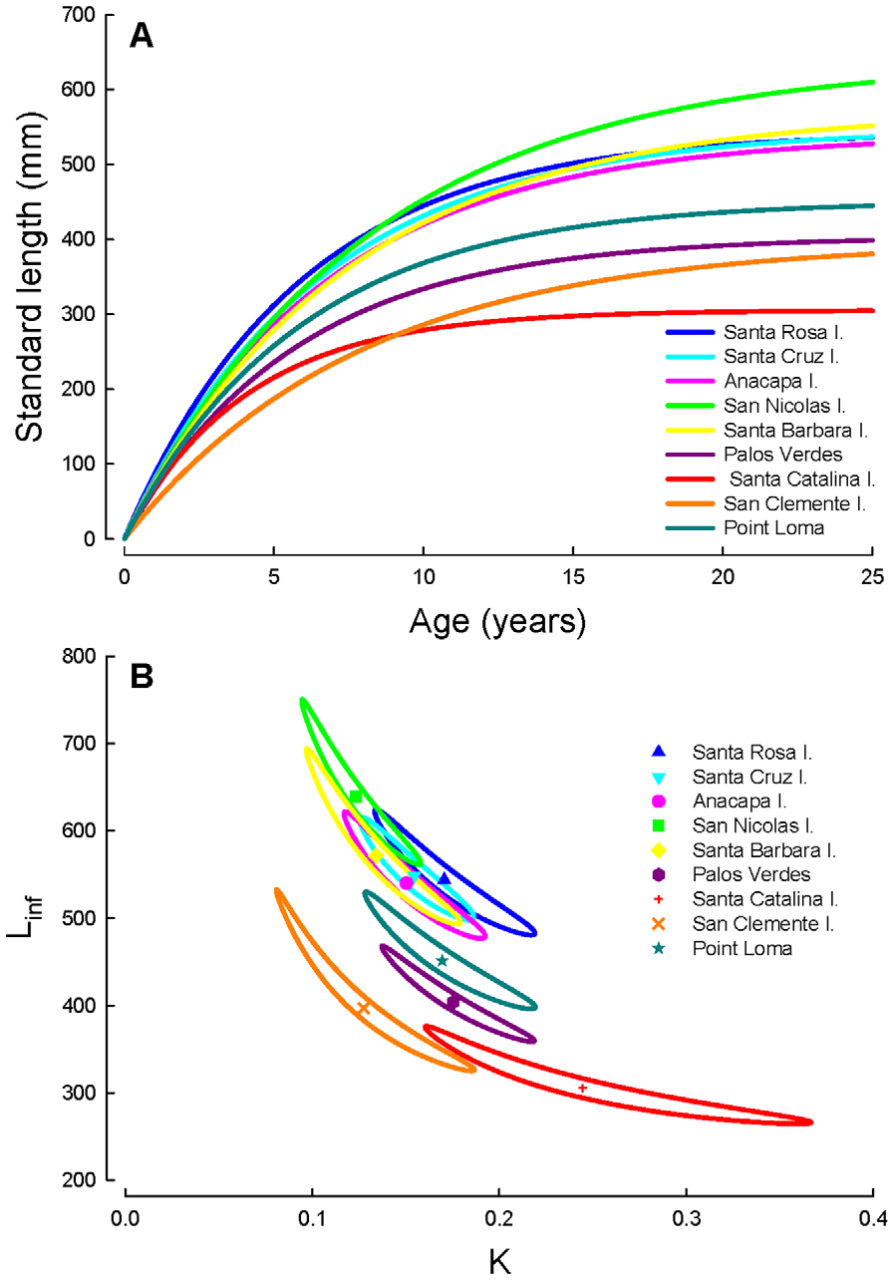
Spatial variation in California sheephead lifetime growth curves across the nine sampled populations. (A) Von Bertalanffy growth curves fit to the size at age data of each population using least squares regression. (B) 95% confidence ellipses around the best-fit parameter values of L_inf_ and K for each population (points), from the Von Bertalanffy growth model.

Lifetime growth curves, estimated from VBGF fits to the size at age data, indicated distinct differences in the growth rates and asymptotic sizes attained by the various southern California populations (Table 1; Fig. 3a). Populations in the northern Channel Islands were largest at age, with fish from the mainland reaching intermediate sizes at age, and fish growing slowest and reaching the smallest sizes at the southern Channel Islands. Differences in growth trajectories among populations were so great that 95% confidence intervals only overlapped for the northern Channel Islands populations, but not those from the mainland or southern Channel Islands, which were significantly different from all other populations (Fig. 3b). Individual VBGF model fits to the size at age data for each population are presented in Fig. S1.

**Figure 4:**
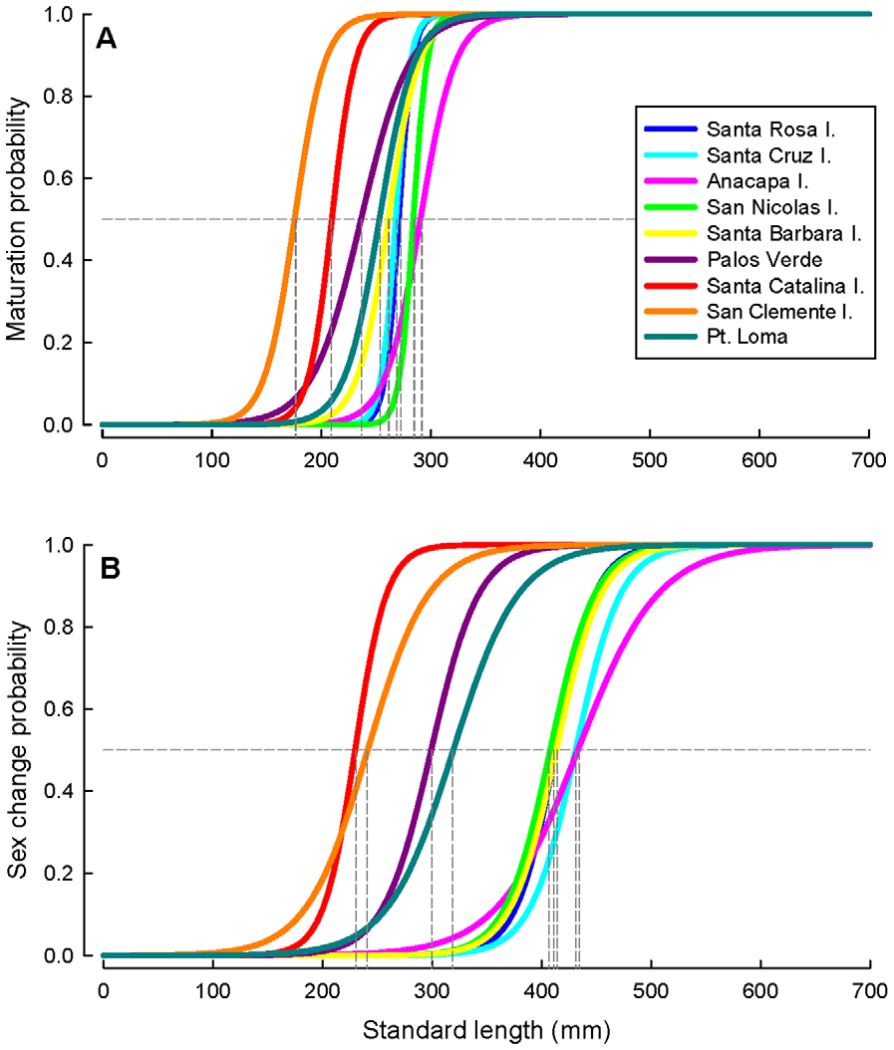
Logistic regression curves predicting (A) the size at maturation and (B) the size at sex change among the nine populations of California sheephead sampled in southern California. Vertical dashed grey lines represent the size of maturation and sex change, defined as the size at which 50% of the population (horizontal dashed line) is a mature female (i.e., maturation) or a mature male (i.e., sex change), respectively.

**Figure 5:**
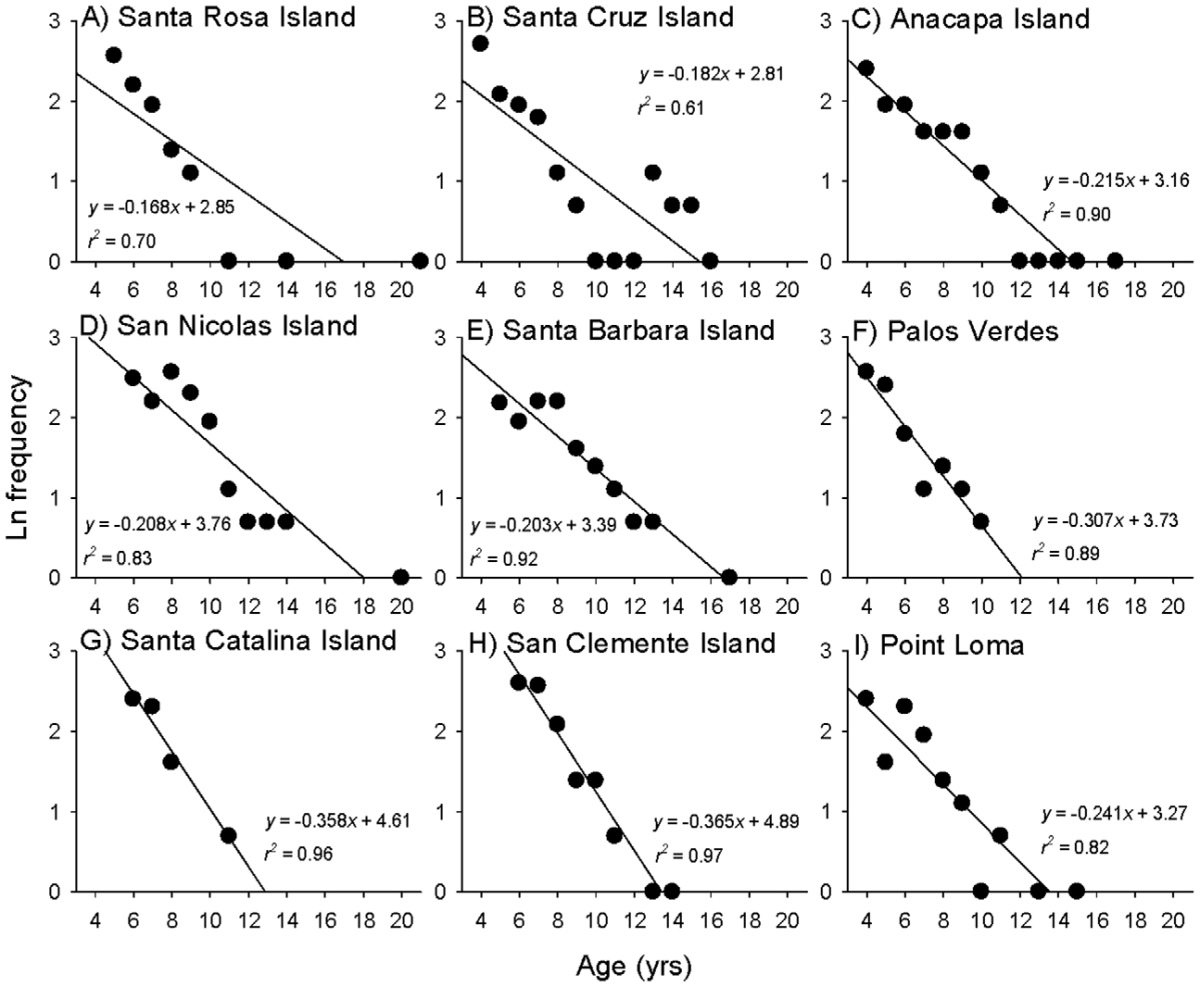
Age based catch curve estimates of instantaneous mortality rates from log-linear regressions of age frequency data for California sheephead across the nine sampled populations. Regression slopes were used to estimate natural mortality rates in the fishery model.

The size at maturation displayed considerable variation among locations throughout southern California, with fish maturing at larger sizes in the northern Channel Islands, compared to the four southern island and mainland populations (Table 1; Fig. 4a). In contrast, the age at maturation showed less variation and all populations matured between 4–5 years of age (Table 1). Differences in growth rates, but similar ages at maturation explain the significant among-site variation in the size at maturation. Both the size and age at sex change differed greatly among locations and three distinct groups were present; fish changed sex largest at the northern Channel Islands, at intermediate sizes at Palos Verdes and Point Loma, and smallest at Santa Catalina and San Clemente Islands (Table 1; Fig. 4b). Confidence intervals around the estimated size and age at maturation and sex change are presented in Table S1. Log-linear regressions of age frequency data revealed differences in mortality and survivorship among locations (Table 1; Fig. 5), although this was marginally non-significant when statistically assessing the differences in slope among locations (ANCOVA: site×age, *F_8, 70_* = 1.9, *P* = 0.07). Annual survival rates were highest at the northern Channel Islands, and lowest at Santa Catalina and San Clemente Islands (Table 1). Survivorship was intermediate at Palos Verdes and Point Loma.

### Incorporating demographic and life history variation into fisheries models

**Figure 6:**
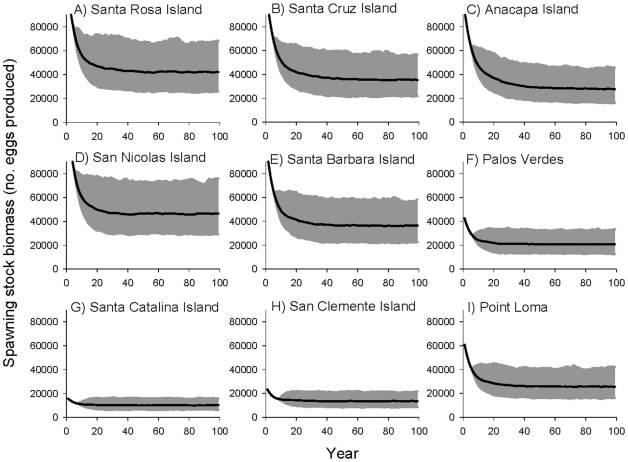
Trajectories of spawning stock biomass (i.e. egg production) at each site from fisheries model projections using the current fishing regulations and fishing mortality rates (**Table 2**) paired with the current demographic and life history parameters (**Table 1**). Each population was initialized with 1,000 age 0 individuals and spawning stock biomass (SSB) was calculated given location specific demographic and life history parameters. Plots show mean trajectories for 1,000 model runs over 100 years (black line) and 95% CI (grey shaded area).

To demonstrate model results, we present the predicted trajectories of spawning stock biomass (SSB) for 100 years, given the current minimum size limit (273 mm SL = 30 cm TL; SL [in mm]  = 0.80*TL+3.23, *r^2^* = 0.99) and location-specific fishing mortality rates calculated as F = Z − M (Fig. 6; Table 2a). Geographic differences in patterns of demography (used to parameterize the model) resulted in consistent differences in predictions of SSB at 100 years. SSB is higher in the northern Channel Islands (Fig. 6a–e) because fish grow more rapidly and attain larger sizes at these sites (i.e., greater maximum asymptotic length [*L_inf_*] from the VBGF; Table 1; Fig. 3). In addition, because fish change sex at larger sizes and older ages in these locations (Table 1; Fig. 4), females spend more years producing eggs before transitioning into terminal phase males. However, given the current demographic and life history parameters, these populations are potentially more vulnerable to increased exploitation because the size at maturation is above the current minimum size limit (273 mm SL) for the fishery (Table 1). In contrast, SSB was lower at the two mainland sites and lowest at the southern Channel Islands (Fig. 6f–i), again, reflecting demography and life history traits of those populations. Fish from these locations attained smaller maximum sizes from estimates of lifetime growth curves and changed sex at smaller sizes and younger ages (Table 1; Figs. 3,4). As a result, average female size was smaller in these populations and individual females spent fewer years producing eggs before changing sex. Consequently, fish at Santa Catalina and San Clemente Islands may be more resilient to increased exploitation because they mature and change sex below the current minimum size limit.

**Table 2:** Minimum size limits, fishing mortality rates, and the median ratio of unfished spawning stock biomass under different management scenarios.

Management type/Population	Minimum size limit (SL, mm)	Fishing mortality rate (*F = Z* − *M*)	SSB/SSB_0_ (±SD)
**A. Minimum size limit optimized, current fishing mortality rate**			
Santa Rosa I.	301	0.068	0.828 (±0.23)
Santa Cruz I.	329	0.082	0.810 (±0.20)
Anacapa I.	331	0.115	0.649 (±0.15)
San Nicolas I.	392	0.108	0.922 (±0.29)
Santa Barbara I.	343	0.103	0.830 (±0.23)
Palos Verdes	303	0.207	0.820 (±0.23)
Santa Catalina I.	236	0.258	0.750 (±0.25)
San Clemente I.	281	0.265	0.840 (±0.21)
Point Loma	292	0.141	0.732 (±0.22)
**B. Current minimum size limit, fishing mortality rate optimized**			
Santa Rosa I.	273	0.145	0.676 (±0.20)
Santa Cruz I.	273	0.161	0.560 (±0.15)
Anacapa I.	273	0.130	0.504 (±0.12)
San Nicolas I.	273	0.161	0.660 (±0.21)
Santa Barbara I.	273	0.161	0.630 (±0.18)
Palos Verdes	273	0.311	0.660 (±0.19)
Santa Catalina I.	273	1.485	0.767 (±0.26)
San Clemente I.	273	0.492	0.772 (±0.21)
Point Loma	273	0.191	0.621 (±0.18)
**C. Local management: both minimum size limit and fishing mortality rate optimized for each population**			
Santa Rosa I.	362	0.266	0.770 (±0.22)
Santa Cruz I.	365	0.251	0.710 (±0.19)
Anacapa I.	367	0.253	0.564 (±0.14)
San Nicolas I.	369	0.206	0.866 (±0.27)
Santa Barbara I.	365	0.251	0.782 (±0.22)
Palos Verdes	308	0.552	0.717 (±0.20)
Santa Catalina I.	241	0.552	0.694 (±0.24)
San Clemente I.	278	0.522	0.777 (±0.21)
Point Loma	281	0.497	0.454 (±0.15)
**D. Regional management: two separate stocks (current MSL = 273 mm; ** ***F*** ** Zone 1 = 0.096, ** ***F*** ** Zone 2 = 0.220)**			
Zone 1 (north): Santa Rosa, Santa Cruz, Anacapa, San Nicolas, Santa Barbara	363	0.219	0.741 (±0.20)
Zone 2 (south): Palos Verdes, Santa Catalina, San Clemente, Point Loma	260	0.235	0.700 (±0.20)
**E. Global management: one stock (current MSL = 273 mm; ** ***F*** ** = 0.119)**			
All populations	324	0.221	0.723 (±0.20)

(A) Minimum size limits for each population that maximize yield given the current fishing mortality rates and the criteria that population biomass cannot fall below 10% of virgin levels in more than 5% of 1000 model simulations. (B) Optimal fishing mortality rates that maximize yield of each population with the minimum size limit fixed at current levels. (C) Simultaneous optimization of minimum size limits and fishing mortality rates given the local demographic and life history parameters for each population. These regulations maximize yield under a local management scenario where each population has independent regulations. (D) Regional management scenario under the assumption that populations can be divided into two separate stocks in southern California. Results show the combination of minimum size limit and fishing mortality rate for each region that maximizes yield while keeping the biomass of all populations in that region above 10% of virgin levels. (E) Minimum size limit and fishing mortality rate that maximizes yield under the global management scenario that assumes populations should be managed as one combined stock.

**Figure 7:**
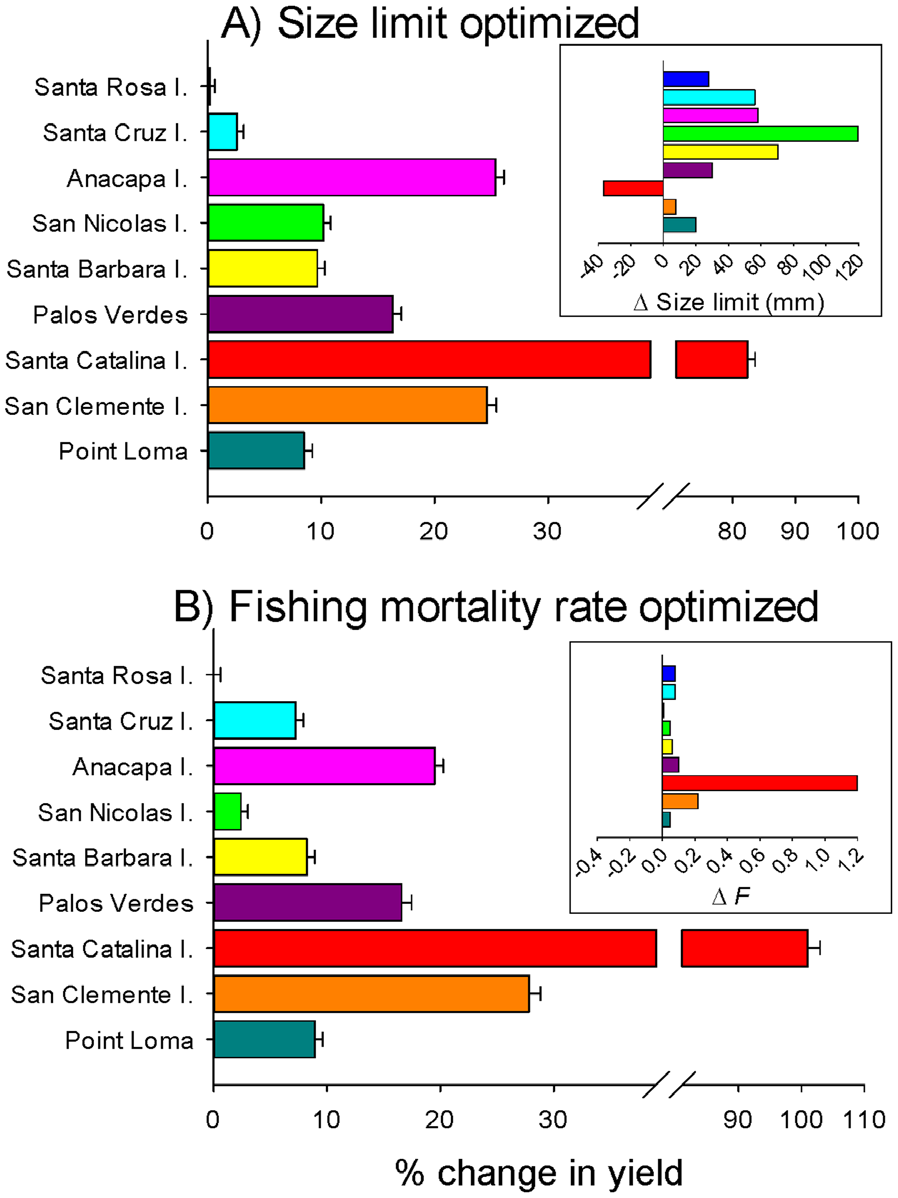
Change in yield when optimizing (A) minimum size limits and (B) fishing mortality rate (*F)*, while keeping the other population parameters constant. Insets show the optimal changes relative to the status quo in (A) size limit and (B) fishing mortality. Bars are mean values ±1 SE.

Our first objective was to evaluate the location-specific yield benefit by optimizing minimum size limits given the demographic and life history parameters for each location, while holding the current fishing mortality rates constant. We compared the median catch at year 100 for simulations parameterized with the optimal minimum size limits to simulations parameterized with the current minimum size limit (Fig. 7a). In searching parameter space for optimal size limits that maximize yield in each population, while adhering to our sustainability criteria (i.e., SSB never drops below 10% of virgin levels in more than 5% of the simulations), we found that potential yield could increase from 2–82% across populations (Fig. 7a). On average, optimizing minimum size limits resulted in an approximately 20% increase in yield. For every population in the northern Channel Islands, increases in yield occurred when the minimum size limit was increased over the current regulations (Table 2a; Fig. 7a inset). At the northern Channel Islands, maximum yield occurred when size limits were raised by 30–120 mm. Yield was highest at Palos Verdes and Point Loma with an increase in the minimum size limit by 30 and 20 mm, respectively, while the San Clemente Island population maximized yield near the current regulations. In contrast, increased yield at Santa Catalina Island occurred when the size limit was reduced by 40 mm; likely a consequence of the small size at maturation and sex change of this population.

We performed a similar analysis to assess how changes in fishing mortality rate could affect fishery yield, independent of size limit. In this instance, we optimized fishing mortality rates while holding the minimum size limit at the current level. Results from this analysis show that location-specific yield can be increased for all populations, by raising fishing mortality rates (Table 2b; Fig. 7b), while still meeting sustainability criteria. Increases in yield from 1–100% occurred across the nine populations, but averaged around 21%. Maximizing yield in this manner, however, required a highly variable increase in fishing mortality rates, from 10–470% over the current levels (Table 2b; Fig. 7b inset). For most populations, relatively large increases in fishing mortality rate (>100% of current levels) were required to attain only a modest increase in yield. Given Santa Catalina Island's demographic and life history parameters and the assumptions of the fishery model, this analysis suggests it could be fished more intensely while conservatively maintaining SSB greater than 10% of virgin levels. This result occurs because fish at Santa Catalina Island mature and change sex below the current minimum size limit and thus have an opportunity to breed successfully, even with greater fishing pressure.

**Figure 8:**
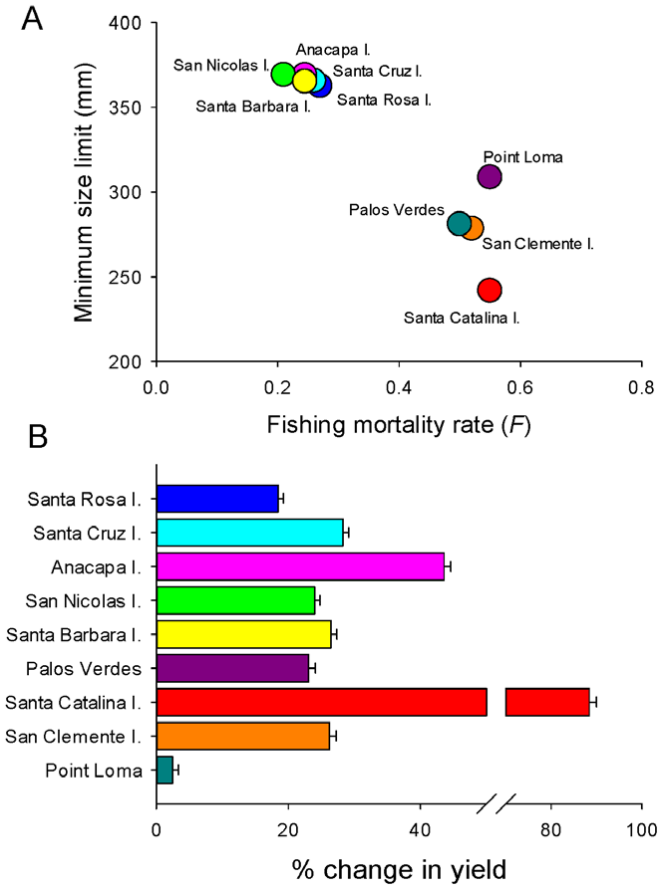
(A) Optimal size limits and fishing mortality rates for each population under the ‘local management’ scenario. (B) Percent change in yield for each population when minimum size limits and fishing mortality rates are simultaneously optimized. Bars are mean values ±1 SE.

We extended this analysis to examine the optimal combination of minimum size limit and fishing mortality rate that achieved the maximum potential yield, given the demographic and life history parameters for each population (Table 2c; Fig. 8a). By optimizing minimum size limits and fishing mortality rates simultaneously, under a local management scenario, potential fishery yield could increase from 2–88% among populations, with an average increase of 31% (Fig. 8b). In general, the northern Channel Islands required an increase in the minimum size limit and modest increases in fishing mortality to maximize yield, while the southern Channel Islands and mainland populations required an increase in fishing mortality rates with modest changes in the minimum size limit (Table 2; Fig. 8a). Given our strict sustainability criteria, most populations maintained relatively high ratios of fished to unfished SSB (Table 2). See the Fig. S2 for plots depicting the equilibrium yield of each population over all plausible combinations of minimum size limits and fishing mortality rates.

**Figure 9:**
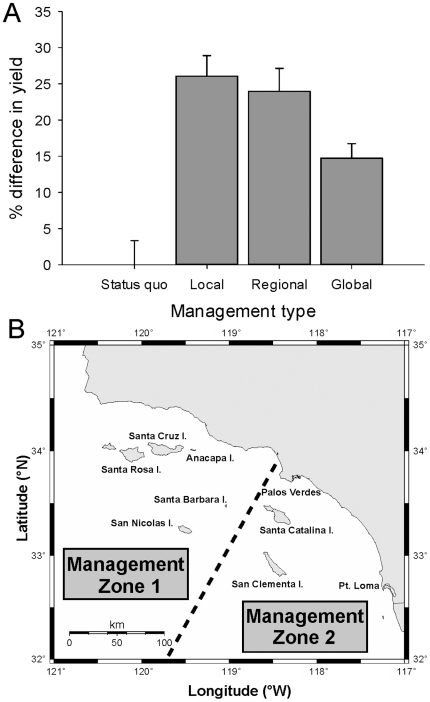
Percent change in yield for the whole California sheephead fishery under four management scenarios. Status quo reflects model output given the current size limit and fishing mortality rates. Local management reflects separate size limits and fishing mortality rates tuned to each population (see Table 2c; Fig. 8a). Regional management assumes two separate stocks with different regulations in the northern and southern regions (see Table 2d). Global management assumes one stock and finds a new optimal size limit and fishing mortality rate while meeting sustainability criteria (see Table 2). Shown are mean cumulative increases in potential fishery yield ±1 SE from 1,000 simulations of the fishery model. (B) Suggested demarcation of two separate California sheephead management zones in southern California according to the grouping of sites that maximizes yield under the ‘Regional Management’ framework.

Overall, cumulative fishery yield could increase by over 26% if each population was managed independently with local regulations governing minimum size limits and fishing mortality rates, compared to the status quo (Fig. 9a). However, this level of local management may be unnecessary because the percent increase in cumulative fishery yield is similar (24%) when populations are assigned to one of two regions with unique size limits and fishing mortality rates (Table 2d; Fig. 9), instead of managed independently. One northern management zone would consist of populations from the northern Channel Islands and in this zone the minimum size limit should be increased by 90 mm over the current regulations in order to maximize fishery yield (Table 2d; Fig. 9b). The southern management zone, comprised of the southern Channel Islands and mainland populations, maximizes cumulative fishery yield near the current minimum size limit. Furthermore, we found that a new global set of regulations with a greater minimum size limit (50 mm larger) and fishing mortality rate (0.102 higher) could increase cumulative fishery yield by approximately 15% over the current management regulations (Table 2e; Fig. 9a).

## Discussion

In southern California, from Point Conception to the Mexican border, we found evidence for extensive spatial variation among nine California sheephead populations in terms of size structure, growth rates, the age and size at maturation and sex change, and annual survivorship. Populations in the northern Channel Islands were composed of larger individuals of all sexual classes, fish were larger in size at age, matured and changed sex at larger sizes, and experienced higher survivorship than populations along the mainland coast south of Los Angeles and at the southern Channel Islands. Previously, spatial variation in demographic and life history traits have been described for this species, but only on larger geographic scales approaching that of the species range [22], [33], [35]. We explicitly incorporated these sources of demographic and life history variation into standard fisheries models for California sheephead and developed a model framework with which to include this type of information in fisheries management decisions. We showed that managing this species on smaller spatial scales, with different minimum size limits and fishing mortality rates tuned to each location or larger region, could lead to increases in fishery yield, while achieving particular conservation objectives.

### Factors responsible for geographic variation in demography and life history

Demographic and life history variation is common in fishes at a variety of spatial scales, from sites within reefs to whole ocean basins [16]–[21], [30], [55] and a multitude of interacting factors are likely responsible for observed patterns. Often, geographic differences in growth rates or the timing of maturation have been related to latitudinal changes in temperature or productivity [e.g., 14]. For California sheephead, fish grow faster and attain larger sizes in colder locations [22], [35], as temperatures are often negatively correlated with productivity (i.e., upwelling) along the west coast of North America. However, this is not strictly a clinal pattern, as upwelling centers occur intermittently along the coast [56]. For example, San Nicolas and Santa Catalina Islands occur at roughly the same latitude, but average water temperatures differ by 3–4°C [57] because San Nicolas Island (where fish are large at age) is bathed by the cool California Current, while Santa Catalina Island (small size at age) is influenced by the warmer Southern California Countercurrent. Demographic and life history variation may also be a density-dependent response in California sheephead as fish from high density populations grow slower and change sex at smaller sizes, after controlling for differences in temperature [22]. Interestingly, the spatial variation in growth rates and the size at maturation and sex change that we observed within southern California is greater than the variation reported previously across larger geographic scales, approaching that of the species' range [22], [33], [35].

Fish populations also respond to the history of exploitation and size-selective fishing has been shown repeatedly to alter demographic and life history traits [23]–[28]. California sheephead are not immune to these effects and intense fishing has been shown to alter size structure and significantly reduce the size at maturation and size at sex change of populations at San Nicolas and Santa Catalina Islands [29]. Spatially variable exploitation by the commercial and recreational fishing sectors throughout southern California [58] has likely left a lasting impact on the current demographic and life history patterns. Demographic and life history traits appear flexible in response to the history exploitation. At San Nicolas Island, where fishing pressure has decreased sharply from the 1998 peak, the 2007–2008 collections indicate a strong recovery has taken place with shifts in life history traits towards pre-exploitation (1980) levels [29, Hamilton et al., *in prep*]. However, where fishing pressure remains high (Santa Catalina and San Clemente), life history traits have not recovered and continue to resemble 1998 levels [22], [29]. For California sheephead, differences in trophic ecology, measured from the same individuals collected for this study, have been shown to correlate with demographic and life history traits [59]. Populations with diets dominated by crabs and sea urchins reached larger asymptotic sizes, matured and changed sex at larger sizes, and experienced higher survivorship, in contrast to populations that consumed higher proportions of bivalves, barnacles and bryozoans. Ultimately, many of these factors are likely to interact so that fish grow faster and attain larger sizes in cooler more productive waters, which enhance the productivity of preferred prey, and in locations where size-selective fishing pressure is lower. These hypotheses fit the current geographic patterns for California sheephead such that fish from the northern Channel Islands live in areas that are generally cooler, more productive, and experience lower fishing mortality rates than sites along the mainland or at the southern Channel Islands, which are warmer, less productive, and closer to population centers near Los Angeles and San Diego, where fishing activities are centered.

### Accounting for geographic differences among populations in fisheries models

The 2004 stock assessment for California sheephead [5] evaluated the effects of spatial variation in demographic and life history parameters on model results, given previous studies indicating that important differences occurred between populations in southern California and central Baja California, Mexico [35]. In southern California, data were only available from Santa Catalina and San Nicolas Islands, prior to heavy exploitation, and these populations appeared to grow at similar rates [29], [33], [35]. Because the stock assessment would only result in management actions for California populations, values from Santa Catalina were ultimately used to parameterize the fisheries models in the stock assessment, despite evidence that demographic and life history parameters from the Baja California populations produced different model results. Here, we parameterized a basic, size- and age-structured population dynamic model with spatially-explicit demographic and life history information from nine southern California locations. We showed that relative fishery yield may be increased by optimizing minimum size limits and fishing mortality rates, while ensuring that populations did not fall below 10% of virgin biomass. Interestingly, for all populations in the northern Channel Islands, increases in yield occurred when minimum size limits were raised over their current level. In general, yield was optimized at southern island and mainland sites when size limits were raised slightly, maintained near current levels, or reduced. The best explanation for this result is that most populations in the northern islands mature and change sex above the current minimum size limit, and therefore heavy fishing pressure leads to the harvest of non-breeding individuals. Optimal minimum size limits were greatest in those locations where fish matured and changed sex at the largest sizes (i.e., the northern Channel Islands). Increases in yield also occurred when fishing mortality rates were raised, given the current size limits. However, in relative terms fishing mortality rates often had to be doubled to achieve increases in yield comparable to those attained by relatively smaller changes in size limits. By managing at local and regional scales, fishing pressure was able to be tailored to the individual and regional population dynamics. When managing at the global scale, our conservative approach for minimizing the probability that a population falls below 10% of unfished spawning stock biomass necessitated management regulations that protect the most vulnerable population across the entire seascape. In effect, this minimized the potential yield of robust populations while maintaining higher than necessary levels of spawning stock biomass in the most vulnerable population.

It has been suggested that spatial variability in nearshore rocky reef resources necessitates a new paradigm in fisheries management [12], [60]. This new paradigm embraces area-based management strategies that account for spatial variability inherent in many nearshore reef species [61], [62]. While still relatively rare, there are examples of a number of fisheries management strategies throughout the world that are tailored to meet the needs of small-scale variation in demographics. For instance, ref. [63] identified the need for spatially explicit management in the Victorian blacklip abalone (*Haliotis rubra*), which exhibits reef-scale differences in growth and maturity. Since 2002, this fishery has utilized harvest policies that adhere to reef-based minimum size limits and quotas. In the state of Washington, Pacific geoduck (*Panopea abrupta*) are managed as multiple populations with rotational harvest of management units occurring every few years [64]. In California, the recent cabezon (*Scorpaenichthys marmoratus*) stock assessment [65] explored the potential for managing the resource at sub-regional scales, but settled on three stock units along the west coast of North America. In 2008, the blue rockfish (*Sebastes mystinus*) stock assessment [66] off western North America identified variability in growth rates as a key prohibitive factor in assessing the population south of Point Conception. The authors recommend spatially explicit data collection programs that account for this variability. In this study, responding to a plea for spatially explicit data, we have set the stage for area-based management through the identification of extreme site-to-site variability in demography of a temperate reef fish. Our simple age- and size-structured model can be applied to many fisheries throughout the world in which spatial variation in demography is observed. To be effective, the benefits of managing at smaller scales must be expressed in simple terms that can inform the optimal management of these resources.

We made a number of model assumptions that could affect our results, however many of these assumptions were made for simplicity or to allow direct comparisons with fisheries models developed previously for California sheephead [5], [42], [43], [50]. First, all populations were initialized with 1,000 age zero individuals in the first year. We recognize the fact that environmental variability may lead to variability in unfished equilibrium conditions, but we suggest that our results will remain qualitatively similar in regards to the strength of adjustments necessary in minimum size limits to effect positive changes in yield. We also assumed a relatively low value for natural mortality (*M* = 0.1), that did not vary temporally nor geographically. Fishing mortality rates were then calculated as the difference between total mortality at a location (measured with catch curve analysis) and natural morality. We are cognizant that our natural mortality estimates are less than that assumed by the stock assessment (*M* = 0.2) [5], but higher estimates often exceeded the total mortality estimates for a given location and the stock assessment estimate was highly uncertain. In our analyses, changes in yield are presented in relative terms and we compare the changes in yield from a given management strategy to the status quo. Both the status quo and the optimal management strategy are calculated from the same parameterization of *M* and other life history variables. Our results are qualitatively similar regardless of the imprecision in natural mortality. Natural mortality is an extremely difficult parameter to estimate, and often confounds stock assessment results, but is unlikely to vary substantially site-to-site in southern California because natural predators are rare. We suggest that future research should focus on utilizing catch curve analyses from no-take marine protected areas or mark and recapture programs to estimate these parameters more accurately.

We assumed that each population was closed with respect to larval and adult transport. Furthermore, the value of the steepness parameter in the stock-recruitment function was assumed to be 0.7 following ref. [5]. It is unknown if this parameter varies geographically and to what extent there is larval connectivity between populations, although previous genetic studies have indicated panmixia between disjunct populations along the Pacific west coast and those in the Sea of Cortez [67], as well as between populations sampled in this study (G. Bernardi, *pers. comm.*). We also assume that density dependence manifests itself at some point between the egg and settlement stage. We make these simplifying assumptions based on the desire to present our results as a general framework for understanding the need to manage at small spatial scales when demographic variability is observed in such dramatic fashion. We also recognize that California sheephead population dynamics may be affected by local social hierarchies, as well as the fishing pressure within a given population, and that maturity, sex change schedules and growth rate parameters may change in response. We do not expressly account for these possibilities in our model, but rather leave the parameters fixed in time and space. In the future, we could make this model more realistic by constructing a metapopulation model with larval dispersal driven by detailed models of ocean circulation [68], initial population sizes scaled to population density and the area of available habitat, as well as incorporation of fishery-induced changes in demographic parameters. Because demographic and life history traits appear to change rapidly in response to the history of exploitation [29], greater realism may be attained in fisheries models by incorporating flexibility in these parameters. However, what remains to be worked out in the future is how quickly demographic and life history traits can recover, if at all, when fishing pressure is alleviated.

### Management recommendations and ecological implications

Local management at the scale of individual populations (i.e., islands in this instance) has the potential to increase total fishery yield by over 26%, while still meeting our sustainability criteria. Practically, however, it would be a logistical challenge for any fisheries management agency to enforce different size or catch limits at this spatial scale. These types of small-scale regulations are likely easier to implement for commercial than recreational fisheries, but for California sheephead, annual landings are comparable for both fishing sectors [5]. However, managing this fishery as two separate regions showed very little decrement in total fishery yield. In this regional management scheme, size limits would be increased by 90 mm in the northern region but left about the same in the southern region (Table 2; Fig 9b), with an increase in fishing mortality rates in both regions. Managing these two fishery regions or stocks could be fairly simple given the obvious spatial demarcation line separating the northern islands from the southern islands and mainland around Palos Verdes. We found that even a simple size limit change (50 mm greater than current), with no spatial dimension to management could increase total fishery yield by 15%. A larger size limit would protect non-reproductive individuals from harvest because most populations in this study mature and change sex at sizes larger than the current minimum size limit. While this management option is likely the simplest to implement, the potential increase in total fishery yield is less than the regional management option. Moreover, this option would likely eliminate fishing opportunities (in the short term) in specific areas, namely Santa Catalina Island. The projected benefits of conservation therefore must be weighed against social and economic objectives of fisheries management before a decision such as this can be made.

Is area-based management logistically practical for fisheries? Some successful fisheries, such as the famous Bristol Bay sockeye salmon fishery are intensely managed on the scale of local tributaries [69]. This fishery is one of the most productive and well-managed fisheries on the west coast, in part because of the spatial variability in life history traits of discrete stocks over relatively small spatial scales and the differential response of those stocks to climatic variation [70]. Territorial user rights fisheries (TURFs) are a type of local management scheme that could benefit by incorporating small-scale demographic and life history knowledge into management decisions. Under this type of management, fishermen are allocated a section of coastline to manage relatively independently and TURFs have proven to be successful in enhancing stocks of targeted species [71]. By collecting data on local demographic and life history parameters, fishermen may be able to optimize yield while ensuring the sustainability of their exclusive fishing zone. In California, this type of management has been suggested for the red sea urchin fishery, which overlaps with the range of California sheephead.

Overall, our results indicate that increasing the minimum size limit for California sheephead could enhance fisheries yield and maintain spawning stock biomass. In addition, findings from a related study we conducted show that increases in size structure of California sheephead may have strong ecological effects on kelp forest communities. By investigating the trophic ecology of this species throughout southern California, we have found that as California sheephead increase in size, their diet contains more and larger sea urchins [59]. Sea urchins destructively overgraze kelp forests under certain environmental and ecological conditions (e.g., low productivity, removal of kelp by storms, overexploitation of predators, ref. [72]). Therefore, any management measure that can both facilitate an increase in California sheephead size structure while maintaining high catch levels could potentially help prevent the formation of urchin barrens by increasing predation pressure on actively foraging sea urchins.

## Supporting Information

Figure S1 **Spatial variation in California sheephead lifetime growth curves across the nine sampled populations.** Shown are size at age plots and fits of von Bertalanffy growth curves for each population using least squares regression. Refer to Table 1 for parameter values.
(TIFF)

Figure S2 **Contour plots of the parameter space showing projected equilibrium yield (kg) of California sheephead from model runs for various combinations of minimum size limit and fishing mortality rate.** Inset legend shading indicates the magnitude of equilibrium yield. White star depicts the current size limit ( = 273 mm SL) and estimated fishing mortality rate (*F*) of each population.
(TIFF)

Table S1 **Size and age at maturation and sex change estimates for California sheephead from logistic regression models.** Shown are the predicted size and age at 50% maturity and sex change, respectively, along with 95% confidence intervals (CI) around those estimates. Due to small sample sizes of immature individuals at some sites, age a maturation confidence intervals could not be calculated.
(DOC)
