# Supplementary figures and images for: Utilizing Spatial Demographic and Life History Variation to Optimize Sustainable Yield of a Temperate Sex-Changing Fish
Source: PLoS One. 2011 Sep 6;6(9):e24580. doi: 10.1371/journal.pone.0024580 (PMC3167858; doi:10.1371/journal.pone.0024580)

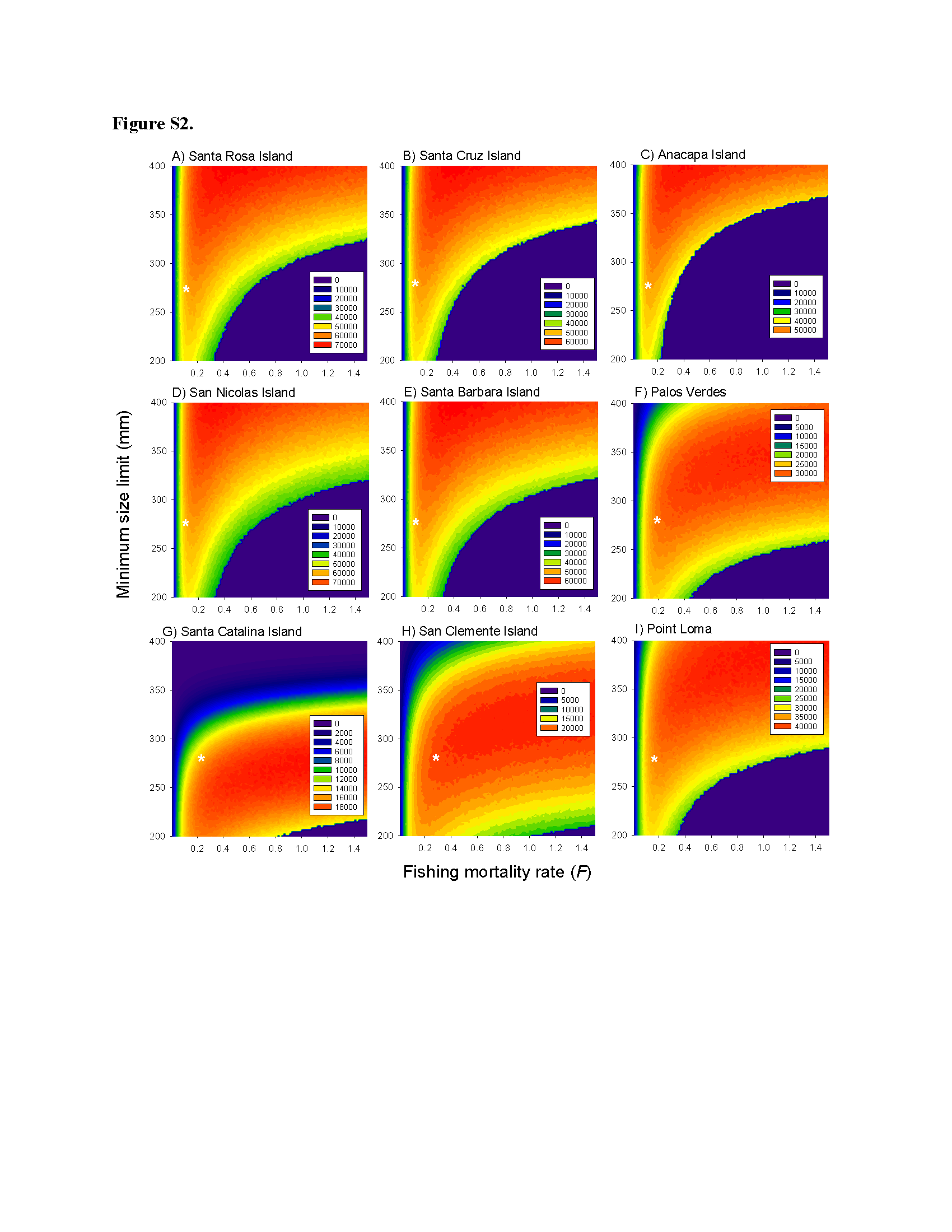

Supplement: Figure S2 — Contour plots of the parameter space showing projected equilibrium yield (kg) of California sheephead from model runs for various combinations of minimum size limit and fishing mortality rate. Inset legend shading indicates the magnitude of equilibrium yield. White star depicts the current size limit ( = 273 mm SL) and estimated fishing mortality rate (F) of each population. (TIFF) [file pone.0024580.s002.tiff]
