# Supplementary material for: Utilizing Spatial Demographic and Life History Variation to Optimize Sustainable Yield of a Temperate Sex-Changing Fish
Source: PLoS One. 2011 Sep 6;6(9):e24580. doi: 10.1371/journal.pone.0024580 (PMC3167858; doi:10.1371/journal.pone.0024580)
Supplement: Table S1 — Size and age at maturation and sex change estimates for California sheephead from logistic regression models. Shown are the predicted size and age at 50% maturity and sex change, respectively, along with 95% confidence intervals (CI) around those estimates. Due to small sample sizes of immature individuals at some sites, age a maturation confidence intervals could not be calculated. (DOC) [file pone.0024580.s003.doc]

**Table S1.**

| Population | Size at maturation | 95% CI | Age at maturation | 95% CI | Size at sex change | 95% CI | Age at sex change | 95% CI |
| --- | --- | --- | --- | --- | --- | --- | --- | --- |
| Santa Rosa I. | 270 | 249-292 | 3.9 | -- | 413 | 389-483 | 8.9 | 7.8-14.8 |
| Santa Cruz I. | 268 | 260-282 | 4.1 | -- | 419 | 400-497 | 11.3 | 9.2-13.7 |
| Anacapa I. | 290 | 270-314 | 4.9 | 4.1-5.8 | 433 | 395-529 | 11.1 | 9.3-16.9 |
| San Nicolas I. | 284 | 271-297 | 4.8 | -- | 408 | 389-427 | 8.4 | 7.7-9.2 |
| Santa Barbara I. | 261 | 217-283 | 4.4 | 2.7-5.0 | 413 | 390-458 | 10.0 | 9.0-12.0 |
| Palos Verdes | 236 | 215-260 | 4.9 | 4.4-5.9 | 299 | 266-365 | 7.7 | 6.2-11.2 |
| Santa Catalina I. | 209 | 81-228 | 4.0 | -- | 230 | 206-249 | 6.2 | 5.0-7.9 |
| San Clemente I. | 175 | NA-199 | 4.9 | -- | 241 | 215-280 | 7.5 | 5.9-9.8 |
| Point Loma | 253 | 226-270 | 4.7 | 4.1-5.4 | 320 | 292-427 | 7.8 | 6.8-10.0 |
|  |  |  |  |  |  |  |  |  |
| Regional and Global population parameters | | | |  |  |  |  |  |
| Zone 1 (north) | 273 | 266-280 | 4.4 | 4.2-4.6 | 414 | 404-425 | 9.6 | 9.1-10.3 |
| Zone 2 (south) | 217 | 205-227 | 4.8 | 4.5-5.0 | 269 | 248-296 | 7.3 | 6.7-7.9 |
| Global (all pop.) | 242 | 233-251 | 4.6 | 4.4-4.7 | 403 | 376-444 | 8.7 | 8.2-9.3 |
